# *Penicillium arizonense*, a new, genome sequenced fungal species, reveals a high chemical diversity in secreted metabolites

**DOI:** 10.1038/srep35112

**Published:** 2016-10-14

**Authors:** Sietske Grijseels, Jens Christian Nielsen, Milica Randelovic, Jens Nielsen, Kristian Fog Nielsen, Mhairi Workman, Jens Christian Frisvad

**Affiliations:** 1 Department of Systems Biology, Technical University of Denmark, DK2800 Kgs. Lyngby, Denmark; 2 Department of Biology and Biological Engineering, Chalmers University of Technology, SE41296 Gothenburg, Sweden; 3 Novo Nordisk Foundation Center for Biosustainability, Technical University of Denmark, DK2800 Kgs. Lyngby, Denmark

## Abstract

A new soil-borne species belonging to the *Penicillium* section *Canescentia* is described*, Penicillium arizonense* sp. nov. (type strain CBS 141311^T^ = IBT 12289^T^). The genome was sequenced and assembled into 33.7 Mb containing 12,502 predicted genes. A phylogenetic assessment based on marker genes confirmed the grouping of *P. arizonense* within section *Canescentia*. Compared to related species, *P. arizonense* proved to encode a high number of proteins involved in carbohydrate metabolism, in particular hemicellulases. Mining the genome for genes involved in secondary metabolite biosynthesis resulted in the identification of 62 putative biosynthetic gene clusters. Extracts of *P. arizonense* were analysed for secondary metabolites and austalides, pyripyropenes, tryptoquivalines, fumagillin, pseurotin A, curvulinic acid and xanthoepocin were detected. A comparative analysis against known pathways enabled the proposal of biosynthetic gene clusters in *P. arizonense* responsible for the synthesis of all detected compounds except curvulinic acid. The capacity to produce biomass degrading enzymes and the identification of a high chemical diversity in secreted bioactive secondary metabolites, offers a broad range of potential industrial applications for the new species *P. arizonense*. The description and availability of the genome sequence of *P. arizonense*, further provides the basis for biotechnological exploitation of this species.

Penicillia are important cell factories for the production of antibiotics and enzymes, and several species of the genus also play a central role in the production of fermented food products such as cheese and meat. An important characteristic of the Penicillia is their ability to produce a large number of structurally and chemically diverse secondary metabolites. These compounds include important pharmaceuticals such as the antibiotic penicillin, the cholesterol-lowering compactin and the antifungal griseofulvin, and the large diversity of bioactive compounds is a valuable reservoir for identification of new pharmaceuticals. In addition, secondary metabolites from Penicillia include mycotoxins, which are frequently found in contaminated food and feed products and pose a health risk to humans and animals. The genus *Penicillium* is large, with more than 354 species that are currently accepted^1^. This fungal diversity is important due to continued demand for biological sources of new enzymes and secondary metabolites^2^ and can lead to the discovery of novel and efficient cell factories for their production.

*Penicillium arizonense* is described here as a new species belonging to *Penicillium* section *Canescentia*. Section *Canescentia* was officially described by Houbraken and Samson^3^ and divides into two clades, one clade containing the well-known species *P. canescens* and *P. janczewskii*, and a second clade containing, *P. atrovenetum* and *P. antarcticum,* amongst others^4^. Members of section *Canescentia* are soil-borne and are characterized by the formation of divaricate biverticillate structures with infrequent additional branches. Phialides are simple and short (7–9 μm) with a broadly cylindrical to slightly or more definitely swollen base and a short, occasionally a more pronounced narrowed neck^3^.

Members of section *Canescentia* are predominantly found in forest litter and soil^5^ and hence possess the natural ability to degrade complex substrates through secretion of a large number of diverse enzymes. Examples of degradative enzyme producers include *P. canescens*, which has been reported to efficiently produce xylanases^6^,^7^ and β-galactosidase^8^, and *P. janczewskii*, which produces high amounts of xylanase, β-xylosidase and α-L-arabinofuranosidase^9^. This natural production of a variety of biomass-degrading enzymes has great potential to be exploited industrially.

The most important secondary metabolite produced by members of section *Canescentia* is the industrial antifungal compound griseofulvin. Apart from *P. griseofulvum*, *P. janczewskii* and the closely related *P. nigricans* were the two subsequent species where griseofulvin was identified^10^,^11^. Later, *P. jensenii* and *P. cansescens*^12^,^13^ have also been identified as griseofulvin producers. Recently, griseofulvin has attracted renewed attention due to reports of complementary bioactivity in mammalian systems, including antiviral and anticancer effects^14^,^15^. Other secondary metabolites identified in *P. janczewskii* include fumagillin^16^ amauromine (nigrifortine)^17^, L-Phe-L-Phe diketopiperazine^18^, MT 81^19^, the antibiotic penicillic acid^12^, cycloaspeptide, pseurotin A^20^ and the indole-diterpenoids pennigritrem and penitrem A^21^,^22^, of which the latter was shown to have tumor suppressant activity in mammary cancer cells^23^. In 1997, patulin and roquefortine C, as well as several analogues and precursors of the latter were reported from *P. janczewskii* isolates^24^, however none of these have been confirmed in *P. janczewskii* or section *Canescentia* since, which also goes for the report of compactins^25^. Curvulinic acid has been identified in both *P. janczewskii* and *P. canescens*, while the antifungal polyketide Sch 642305 has only been detected in the latter^26^,^27^. Other secondary metabolites reported in *P. canescens* are pseurotin A, the tetrapetide D-Phe-L-Val-D-Val-L-Tyr and aurantiamine^28^, however the chemical profile of this strain indicates that it could have been *P. aurantiogriseum* rather than *P. canescens*^1^,^29^. From the series, the ex-type culture of *P. canescens* was reported to produce the oxalicins and decaturin^30^, while the antimicrobial canescin was reported from in 1953 from *P. canescens*^31^. The isolate of *P. janczewskii* reported to produce penigequinolones and gliovictin^32^,^33^ may have been *P. scabrosum*, as these compounds are consistently produced by the latter species^34^.

To fully exploit the biosynthetic capacity of filamentous fungi for the development of novel cell factories, the availability of full genome sequences is of great importance. This allows for the determination of potential biosynthetic capabilities including the identification of secondary metabolite biosynthetic gene clusters. Additionally, the genome sequences enable a knowledge based approach for the optimization of cell factories e.g. through metabolic network modelling, for the production of enzymes or secondary metabolites. Metabolic engineering strategies have previously been used with great success to redesign metabolic fluxes for secondary metabolite production in filamentous fungi^35^,^36^,^37^ and for heterologous expression^38^,^39^,^40^. Enabling the transfer of genes or gene clusters to well-known expression hosts serves as an important step in the development of efficient cell factories that can be used for economically viable production of secondary metabolites in the food and pharmaceutical industry^41^. Heterologous expression of secondary metabolites has been amongst others successfully demonstrated in the yeasts *Saccharomyces cerevisiae*^42^, in the filamentous fungi *P. chrysogenum*^43^, *Aspergillus nidulans*^44^, and *A. oryzae*^45^,^46^.

Here we describe the isolation and genome sequencing of *P. arizonense*. The genome sequence is the first publicly available genome within section *Canescentia*, and hence serves as the first genomic insight into this interesting section. A functional analysis gave insights into the capacity for this species to produce degradative enzymes, which could have biotechnological implications and highlight the potential applications for *P. arizonense* as a source of industrial enzymes. We further identified putative secondary metabolite gene clusters and by coupling this with measurement of secondary metabolites we obtained valuable information on the capacity for secondary metabolite production by this species.

## Results & Discussion

### Genomic features

The genome of *P. arizonense* CBS 141311^T^ = IBT 12289^T^ was shotgun sequenced using illumina 2500 technology (125 bp PE reads) to an approximate coverage of 154 fold. The short reads where assembled *de novo* into 396 contigs longer than 200 bp, 43 supercontigs longer than 100 kb, and the longest contig being 2.7 Mb. This resulted in a cumulative assembly length of 33.7 Mb and a contig N50 of 1 Mb. A total of 12,502 putative genes were identified in the genome including 173 tRNAs. Protein coding genes were functionally annotated by blasting CDS regions at the amino acid level against Uniprot, and 8269 proteins could be mapped to a known homolog. Quality control of the gene prediction was performed by Benchmarking Universal Single-Copy Orthologs (BUSCO)^47^, which assess the completeness of genomes by detecting the presence of 1438 ubiquitous single copy eukaryotic genes. All 1438 genes were found, with only 6 of them being in fragmented versions. The predicted genes covered 59.8% of the assembled genome and the majority of the genes contained at least one intron (84%) with the average number of introns being 2.2 per gene. The GC content of the assembled genome was 49.1%, which is similar to that of related species (Supplementary Table S1). Mitochondrial DNA was identified as a 28,347 bp linear DNA fragment, with a GC content of 25%, showing high similarity to the published mitochondrial genome of the somewhat related species *P. solitum*^48^. A total of 43 mitochondrial genes were identified including 23 tRNA genes and two ORFs orthologous to uncharacterized ORFs in the mitochondrial genome of *P. solitum*^48^. General genomic features of *P. arizonense* are summarized in Table 1.

### Phylogenetic analysis

**Figure 1:**
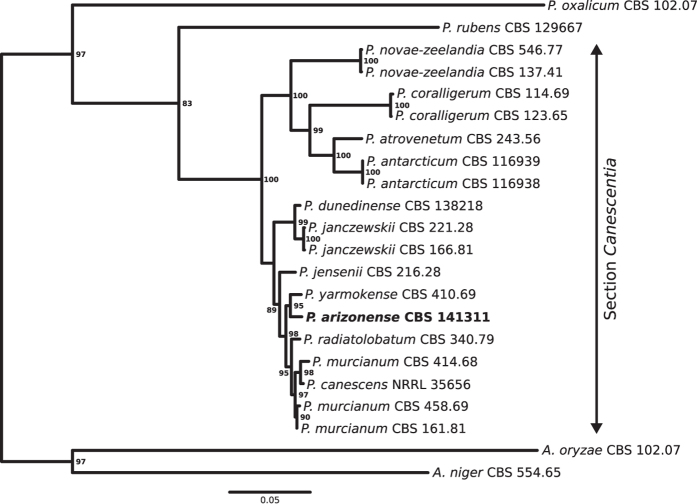
Maximum likelihood phylogram of section *Canescentia* and related species. The phylogram is based on partial RPB2, *camA*, *benA* and ITS sequences, and bootstrap values are given as percent of 1000 maximum likelihood trees. Only bootstrap support of more than 80% is shown.

Partial nucleotide sequences of RPB2, calmodulin (*camA*), β-tubulin (*benA*) and the ITS sequence, from section *Canescentia* and related species were aligned, trimmed and concatenated to a final sequence of 1961 nucleotide positions, to infer a maximum likelihood phylogenetic tree (Fig. 1). The topology of the phylogram was in agreement with previous observations, grouping section *Canescentia* into two major clades^4^. *P. arizonense* grouped within the clade containing the well described species *P. canescens* and *P. janczewskii*, and the closest relative among the tested species was *P. yarmokense*, which represented a sister species with 95% bootstrap support.

### Functional annotation

**Figure 2:**
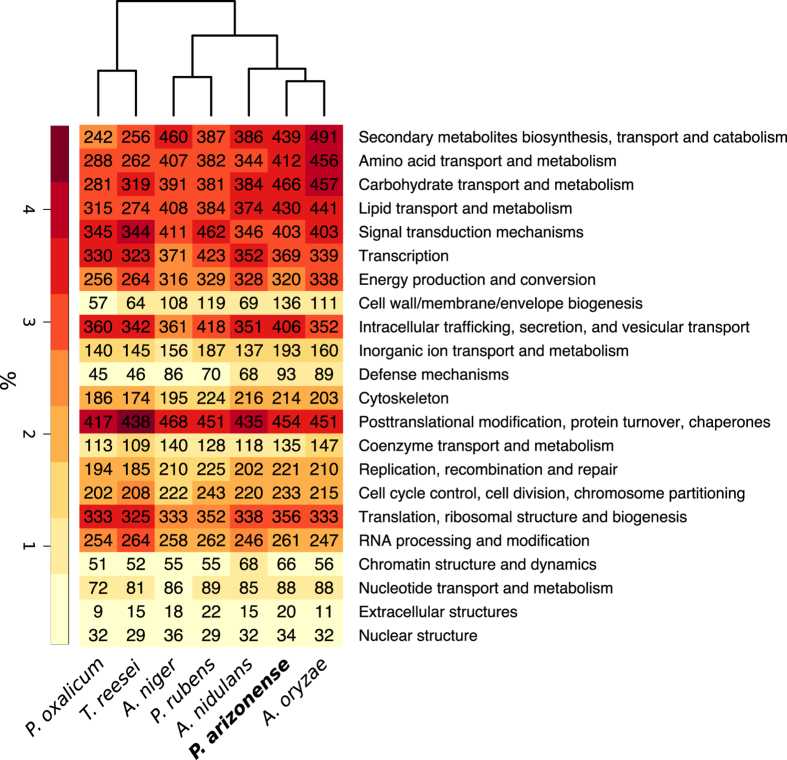
KOG (euKaryotic Orthologous Group) classification of *P. arizonense* and six related fungi. The colors represent percentage out of total number of proteins identified in the genome, and the absolute number of proteins is given as labels.

All proteins of *P. arizonense* were compared at a functional level to 6 related, genome sequenced fungi, selected either as being model organisms and/or because of their ability to secrete carbohydrate active enzymes (*P. rubens* (often identified as *P. chrysogenum*), *P. oxalicum*, *A. niger*, *A. nidulans*, *A. oryzae*, and *Trichoderma reesei*). Using KOG^49^ (euKaryotic Orthologous Group) classification, we assigned functions to proteins based on sequence similarity, to determine the proportions of the proteomes allocated to different cellular functions (Fig. 2). The global pattern of protein allocation in *P. arizonense* was most similar to that of *A. oryzae*, as determined by the clustering, indicating that their ecological niches might be similar. In absolute numbers, *P. arizonense* had the highest number of proteins in the three categories, (*i*) “carbohydrate transport and metabolism”, (*ii*) “cell wall/membrane/envelope biogenesis” and (*iii*) “inorganic ion transport”.

The high number of proteins involved in carbohydrate metabolism in *P. arizonense* correlates with the fact that members of section *Canescentia* are known to thrive in decaying plant material where there is likely a requirement to degrade diverse and complex carbon sources. *P. arizonense* contains more genes involved in carbohydrate metabolism than any of the other species we used for the comparison, including *A. oryzae* which is known to have a diverse arsenal of carbohydrate active enzymes^50^ and is applied for the production of industrial enzymes^51^. This highlights the potential for using *P. arizonense* for biotechnological applications. The value of further studying the genomics of *Penicillium* section *Canescentia* is thus emphasized, in order to elucidate whether this section as a whole is rich in carbohydrate metabolic genes or if this feature is specific to *P. arizonense*.

Furthermore, the KOG analysis showed that *P. arizonense* contains a high number of genes related to “Inorganic ion transport” which suggest that it could be an important species in the nutrient cycle in agricultural settings, where fungi are known to take up and convert inorganic phosphate to more readily accessible organic forms needed by plants. This is of major agricultural relevance since phosphate availability often is the limiting accessible nutrient in soil^52^. It was also noted that *P. arizonense* contains a large number of genes involved in secondary metabolism and lipid metabolism.

### Carbohydrate active enzymes

**Figure 3:**
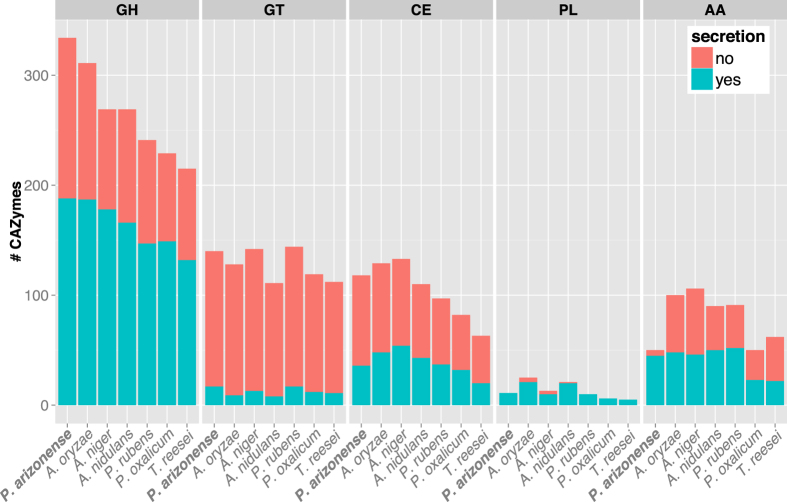
Annotation of secreted and non-secreted CAZys in *P. arizonense* and six related fungi. CAZymes are grouped into the classes: glycoside hydrolases (GH), glycosyl transferase (GT), carbohydrate esterases (CE), polysaccharide lyases (PL) and auxilliary activities (AA).

In order to further investigate the enriched carbohydrate metabolism of *P. arizonense*, Carbohydrate Active enZymes (CAZys) were annotated and their secretion evaluated in the 7 species mentioned above (Fig. 3). In agreement with the KOG analysis showing that *P. arizonense* had a large number of carbohydrate metabolic genes, the total number of CAZymes in the species was also among the highest (668 CAZymes), only exceeded by *A. oryzae* (675 CAZymes). From the distribution of CAZy classes, the large number of CAZymes in *P. arizonense* could mainly be attributed to having a high abundance of glycoside hydrolase (GH) enzymes with 331 in total, 23 more than *A. oryzae* and 65 more than *A. niger* and *A. nidulans* (Fig. 3). The number of GH enzymes containing a secretion signal sequence was also the highest (188), although *A. oryzae* had a similar number (187).

For the carbohydrate degrading CAZy classes, polysaccharide lyases (PL), carbohydrate esterases (CE) and the glycosidic bond forming glycoside transferases (GT), *P. arizonense* had similar abundance as the other species both in total number and in number of enzymes containing secretion signals. In terms of auxiliary activity (AA) CAZymes, which are redox enzymes that work in conjunction with CAZymes aiding access to carbohydrates in plant cell walls^53^, *P. arizonense* contained the fewest number of enzymes together with *P. oxalicum*. However, for *P. arizonense,* the majority of these (90%) contained a secretion signal, where for the other species only 35–55% of the AA CAZymes were annotated as secreted. How this lack of intracellular AA CAZymes affects the physiology of *P. arizonense* is unknown.

Lignocellulose degrading enzymes such as cellulases, hemicellulases and pectinases are of industrial importance for a range of applications, e.g. biomass degradation for production of biofuels, and these enzymes are mainly harboured within the GH class of CAZy. Some of the GH families were widely abundant in all species e.g. GH3 and GH18, and some were unique to a single species. *P. arizonense* contained no unique GH families, however GH29, GH39 and GH42 were seen in *P. arizonense* and each of these were found in only one other species, *A. niger*, *A. nidulans* and *P. rubens*, respectively. In the families GH1, GH65 and GH93, *P. arizonense* had a considerably higher number of enzymes than found in the compared species (see Supplementary Fig. S1). These GH families include enzymes with various catalytic activities i.e. GH1 contains mainly β-glucosidases and β-galactosidases, GH65 contains enzymes with diverse activities, but mainly phosphorylases, and GH93 contains exo-α-L-1,5-arabinanases. Among these enzymes, exo-α-L-1,5-arabinanases are of relevance for biomass conversion since they take part in the degradation of hemicellulose, and is of interest due to their potential rate limiting role in the degradation of lignocellulose^54^.

CAZy families specifically containing enzymes with degradative activity towards cellulose, hemicellulose and pectin were additionally quantified (see Supplementary Fig. S2). *P. arizonense* proved to contain a high number of total hemicellulases (53) together with *A. oryzae* (57), compared to the other species. The number of cellulase and pectinase encoding genes in *P. arizonense* was similar to the other species. Our results correlate with previous findings showing that closely related *P. janczewskii*, efficiently produces hemicellulose degrading enzymes, but possesses only little cellulase activity^55^. This feature is of interest for some processes where cellulases are unwanted, such as pulp processing, and our results indicate that it could be a property shared by *P. arizonense*.

### Secondary metabolite biosynthetic gene clusters and CAZy genes

The genome of *P. arizonense* and 9 related *Penicillium* species were mined for putative secondary metabolite biosynthetic gene clusters using antiSMASH^56^ (Supplementary Table S2). The total number of gene clusters in the species were highly variable ranging from 70 in *P. expansum* to 38 in *P. digitatum*, while the well-known industrially used penicillin producer, *P. rubens*, had 53. In comparison, *P. arizonense* contained a relatively high number with a total of 62 detected gene clusters (Supplementary Data S1), including 28 polyketide synthase (PKS) clusters, 16 non-ribosomal peptide synthase (NRPS) clusters, 5 terpene clusters, 3 hybrid PKS:NRPS clusters, 1 indole cluster and 1 siderophore. In particular, *P. arizonense* contained more PKS clusters than any of the compared species (Supplementary Table S2).

**Figure 4:**
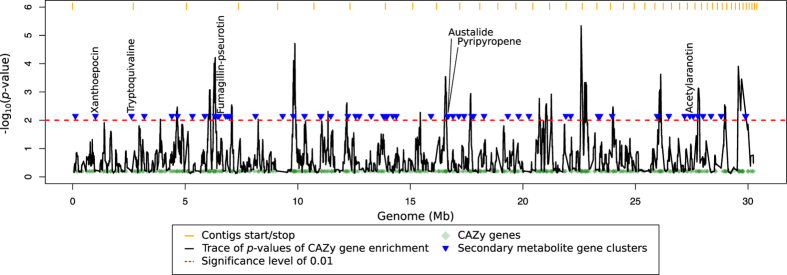
Localization of CAZy genes and secondary metabolite gene clusters in the *P. arizonense* genome assembly. CAZy gene enrichment is given as the p-value of a hypergeometric test in a sliding window of 100 kb and a step size of 10 kb. The genome assembly is visualized by concatenating supercontigs (more than 100 kb), ordered by size.

In *T. reesei*, CAZy genes have previously been shown to cluster into defined regions of the genome, and several of the regions enriched in CAZy genes also contained genes encoding for enzymes involved in secondary metabolism^57^. Since *P. arizonense* has a high abundance of both secondary metabolic gene clusters and CAZy genes, we assessed the genomic localization of these (Fig. 4), using a sliding window approach. A total of 23 genomic regions containing a significant enrichment of CAZy genes (*p*-value <0.01, hypergeometric test) were found in the genome. By superimposing these regions with the predicted secondary metabolic gene clusters, it was observed that 9 CAZy enriched regions were overlapping with secondary metabolic gene clusters. Similarly, Martinez *et al.*^57^ identified 28 enriched CAZy gene regions in *T. reesei*, where 5 of these contained either a PKS or an NRPS^57^. Although most secondary metabolite gene clusters do not clusters with regions enriched in CAZy genes in both *P. arizonense* and *T. reesei*, several overlaps were seen (Fig. 4). Among the co-localized secondary metabolite gene clusters we found in *P. arizonense*, one was predicted to encode the synthesis of austalides and another pyripyropenes (see section below and Table 1). It is intriguing to speculate whether these secondary metabolites could be regulated commonly with the CAZy genes in the region, and if their products have synergistic effects.

### Secondary metabolites

**Figure 5:**
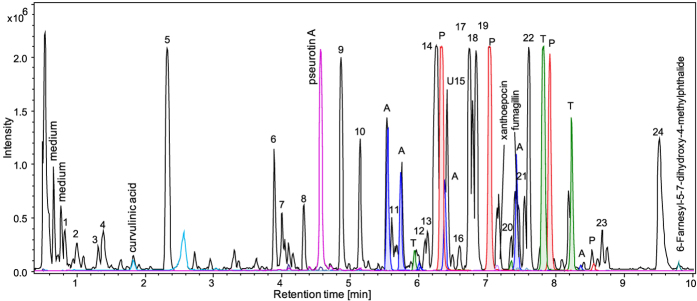
Base peak chromatogram and extracted ion chromatograms of extract of *P. arizonense* grown on CYA medium. Black: base peak chromatogram, blue: extracted ion chromatograms of molecular ions of austalides (A), red: extracted ion chromatograms of molecular ions of pyripyropenes (P), green: extracted ion chromatograms of molecular ions of tryptoquivalines (T). Major peaks that could not be identified: 1–24.

To further investigate the identified diversity in secondary metabolite gene clusters at a chemical level, *P. arizonense* was, after a pre-screening (data not shown), grown on three different solid media and analyzed using liquid chromatography combined with UV/Vis spectroscopy and high resolution mass spectrometry for secondary metabolite profiling. Seven different compounds or families of compounds were identified in the crude extract of *P. arizonense* (Table 2, Supplementary Table S3, Supplementary data S4 and Supplementary Fig. S3). The major peaks identified, belonged to the families of pyripyropenes, austalides and tryptoquivalines. Except for curvulinic acid, pseurotin A and fumagillin, none of the secondary metabolites identified in the extract had previously been found in section *Canescentia*. The compounds compactin, roquefortines, mycelianamide, penigequinolones, chrysogines, and oxalicins/decaturins could not be chemically detected in *P. arizonense*, even though they have been reported to be produced in other species in section C*anescentia*. Many of the peaks in the chromatogram could not be matched with any compounds in the database Antibase (Fig. 5) (Wiley-VCH, Weinheim, Germany), and could be interesting targets for isolation and structure elucidation by NMR. Based on homology to known gene clusters, the predicted clusters in *P. arizonense* were connected to the detected compounds when possible (Table 2 and Supplementary Data S2). All compounds except curvulinic acid could putatively be connected to a predicted secondary metabolite gene cluster in the genome.

#### Austalides

A total of six austalides were identified based on accurate mass, UV/Vis, and very similar MS/HRMS spectra with *m*/*z* 207.065 dominating. The F J isomer could be unambiguously identified by comparison to reference standards, while the remaining five major austalides were only tentatively identified. The austalide gene cluster was not previously described in the literature, but one of the predicted gene clusters in the genome of *P. arizonense* showed similarity to the mycophenolic acid gene cluster of *P. brevicompactum*^58^. Mycophenolic acid is a meroterpenoid consisting of an acetate-derived phthalide nucleus and a terpene-derived side chain. This phthalide structure is also present in the austalides and it is therefore likely that mycophenolic acid and austalides have similar biosynthetic genes. In a putative austalide gene cluster in *P. arizonense,* orthologs were found to the genes *mpaC*, *mpaD*, and *mpaA* from the mycophenolic acid cluster in *P. brevicompactum*, corresponding to the genes responsible for the biosynthesis of the phthalide intermediate, 6-farnesyl-5,7-dihydroxy-4-methylphthalide^58^. This phthalide was also identified in the extract of *P. arizonense*, and confirmed by comparison of HRMS, UV/Vis and MS/HRMS to a reference standard. The austalides are a family of related meroterpenoid metabolites that were isolated for the first time from maize cultures of *A. ustus*^59^. Recently, it was reported that they also were produced by *P. thomii* KMM 4645 and *P. lividum*^60^.

#### Pyripyropenes

Four compounds with accurate masses and UV spectra corresponding to the family of pyripyropenes were detected; pyripyropene E, F, O, and A. The latter was further confirmed by comparison of retention time and tandem MS (MS/HRMS) spectra to a reference standard. The other pyripyropenes had UV spectra and MS/HRMS fragmentation patterns similar to pyripyropene A. The biosynthetic gene cluster of pyripyropene was identified in *A. fumigatus*^45^ and have later been described in *P. coprobium* as well^29^,^61^. One of the predicted PKS clusters in *P. arizonense* contained orthologous genes to 7 of the 9 cluster members of the *A. fumigatus* pyripyropene gene cluster. For the two missing genes the blast analysis suggested a fusion of the *A. fumigatus pyr1* and *pyr2* as well as *pyr4* and *pyr5* in the *P. arizonense* cluster, and this would correspond to a full conservation of the gene order in the two clusters. Pyripyropenes were first isolated from *A. fumigatus*^62^ and later they were found to also be produced by *P. reticulisporum*^63^, *P. coprobium*, *P. concentricum* and *P. coprophilium*^29^. Pyripyropenes have attracted interest since they are highly selective toward, Acyl-CoA:cholesterol acyltransferase, a potential therapeutic target for the treatment or prevention of hypercholesterolemia and atherosclerosis^64^, and showed potent anti-proliferative activity against Human Umbilical Vein Endothelial Cells (HUVECs)^65^. It is interesting to note that while *P. arizonense* produces pyripyropenes, the closely related species *P. canescens* produces the chemically related oxalicins/decaturins^30^.

#### Tryptoquivalines

The presence of a tryptoquivaline with elemental composition C_29_H_30_N_4_O_7_ was confirmed with retention time, accurate mass and MS/HRMS matching the reference standard. This elemental composition corresponds with tryptoquivaline C and 27-epi-tryptoquivaline, however because no stereochemistry was provided by the manufacturer of the standard, they could not be distinguished. Three other peaks had UV spectra and fragmentation patterns similar to the tryptoquivaline standard. However, there are several tryptoquivalines with the same elemental compositions and with only a limited number of tryptoquivaline reference standards; these compounds could only be identified as members of the tryptoquivaline group. The closely related compounds, tryptoquialanins, have been identified in *P. digitatum*^66^ and the gene cluster was described in *P. lanosocoeruleum* (formerly *P. aethiopicum*)^67^. A predicted NRPS cluster in *P. arizonense* was highly similar to the tryptoquialanin cluster of *P. lanosocoeruleum* and contained orthologs of all 13 biosynthetic genes, as well as conservation of the gene synteny except for two genes which have swapped position. Tryptoquivalines are tremorgenic and were first identified in *Aspergillus* species^68^,^69^ and later in *P. jamesonlandense*^70^.

#### Fumagillin and pseurotin A

The presence of fumagillin in the extract of *P. arizonense* was confirmed by comparison of HRMS, UV/Vis and MS/HRMS to a reference standard. It has been shown that the biosynthetic genes of fumagillin are, together with the genes of the compound pseurotin A, located in an intertwined supercluster in *A. fumigatus*^71^. Based on a comparison of HRMS, UV/Vis and MS/HRMS to a reference standard, pseurotin A was also confirmed in the *P. arizonense* extract. A single gene cluster highly similar to the intertwined fumagillin-pseurotin gene cluster in *A. fumigatus*, was found in the genome of *P. arizonense*, encoding orthologs of all enzymes for the fumagillin and pseurotin A biosynthesis. The gene synteny was also fully conserved in the two clusters including 4 co-localized genes that have not been connected to the biosynthesis of fumagillin or pseurotins so far. Similar intertwined fumagillin-pseurotin clusters have been seen in closely related *A. fischeri* (formerly *Neosartorya fischeri*) and in the more distantly related *Metarhizium anisopliae*, but with a less conserved gene organization compared to *A. fumigatus* and *P. arizonense*. Interestingly, several other members of section *Canescentia* produce fumagillin, but only *P. janczewskii* has been reported to produce pseurotin A^20^ as well, suggesting that it also might have the intertwined cluster. This finding suggest that an evolutionary pressure exists in some species to keep the biosynthetic genes for fumagillin and pseurotin A co-localized, possibly related to their co-regulation, which is governed by the LaeA regulated transcription factor FapR in *A. fumigatus*^71^. Fumagillin was first identified in *A. fumigatus*^72^ and later it has been seen in *P. scabrosum*^12^ *P. jensenii*, *P. nigricans*^73^, *P. janczewskii*^16^, all belonging to section *Canescentia*, as well as an undescribed species also allocated to section *Canescentia*^74^. It has antibiotic and antifungal activity and it was also discovered to have anti-cancer properties^75^. Pseurotin A is a heterospirocyclic secondary metabolite originally isolated from *Pseudeurotium ovale*^76^. Pseurotin A has been found to induce cell differentiation of PC12 neuronal cells, highlighting the compound as a potential tool for studying the mechanism of neurite formation^77^. The production of immunoglobulin E (IgE) is suppressed by pseurotin A, suggesting its use as an interventional tool for studies of IgE-mediated systemic allergic response mechanisms^78^.

#### Xanthoepocin

For xanthoepocin there was no analytical standard available but previous dereplication, MS/HRMS fragmentation and the distinctive UV spectrum, previously described by Igarashi *et al.* (2000) (see Supplementary Fig. S4), showed its presence in the extract. The xanthoepocin cluster is not known, but a cluster of the related compound, aurofusarin have been shown to consist of 11 genes in *F. graminearum*^79^,^80^. One *P. arizonense* PKS cluster contained orthologs of 7 of these genes including the PKS, and hence might be responsible for the xanthoepocin biosynthesis. Xanthoepocin has antibiotic activity and was previously identified from *P. simplicissimum*^81^ and *P. excelsum*^82^.

#### Curvulinic acid

The presence of curvulinic acid was confirmed by comparison of HRMS, UV/Vis and MS/HRMS to a reference standard. The gene cluster for curvulinic acid is not described in the literature and amongst the 28 putative PKS clusters found in *P. arizonense* it was not possible to assign any of them to the curvulinic acid biosynthesis. Curvulinic acid was previously shown to be produced by another member of section *Canescentia*, *P. canescens*^26^.

In addition to the detected compounds, a predicted NRPS cluster in *P. arizonense* showed similarity to an acetylaranotin gene cluster from *A. terreus*, with orthologs of all 9 genes^83^. Acetylaranotin is a epipolythiodioxopiperazine, which is a class of secondary metabolites containing di-or polysulfide bridges. None of the peaks in the extracts of *P. arizonense* showed isotopic patterns consistent with sulphur, hence, this putative acetylaranotin cluster is assumed to be silent under the conditions tested in this study.

### Taxonomy

*Penicillium arizonense* Frisvad, Grijseels and J.C. Nielsen, sp. nov.

#### Mycobank MB 817128

Type: Herb. C-F-101845, cultures ex type IBT 12289 = CBS 141311, from a sample of dry red soil, south rim of Grand Canyon, Grand Canyon Village, Arizona, USA (36°, 3′22.31″ N; 112° 7′30.73″ W), Per V. Nielsen, July 1990, fungus isolated by dilution plating by J.C. Frisvad; additional cultures: IBT 12285 = CBS 141312; IBT 12287 = CBS 141313 from the same source as the culture ex type, but not of the same clone.

#### Morphological description

##### Macromorphology

**Figure 6:**
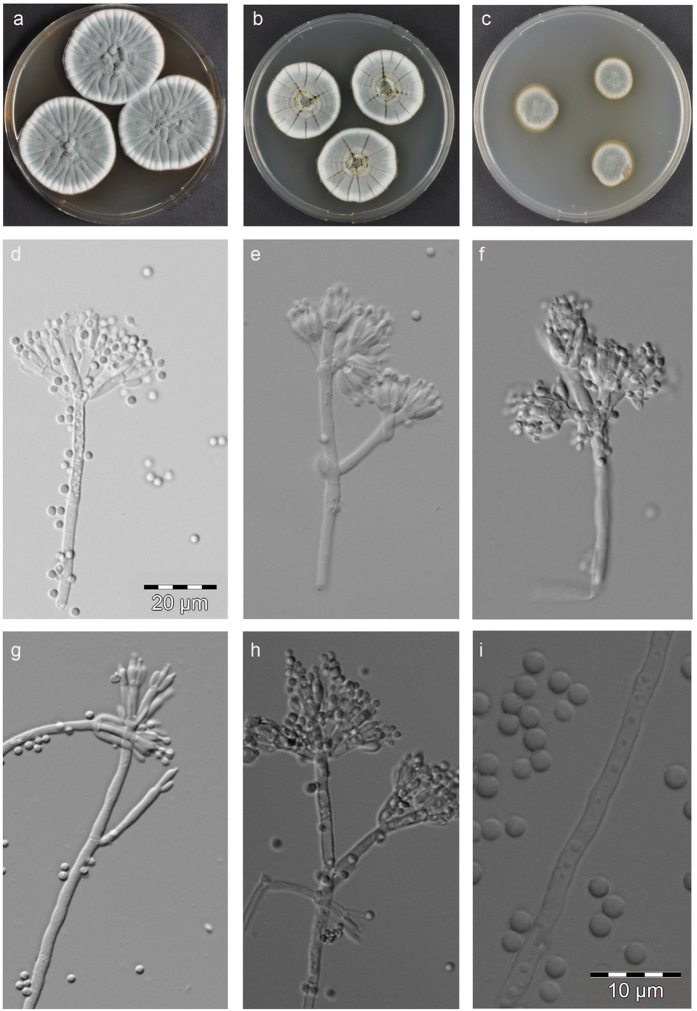
Micro-and macro morphology of *P. arizonense.* Colonies after one week (**a**) CYA (**b**) YES (**c**) MEA, (**d–h**) conidiophores (**i**) conidia.

Colony diameter on CYA agar (Czapek Yeast Autolysate agar) after one week in darkness at 25 °C: 28–35 mm, MEA (Malt Extract Agar): 11–29 mm, MEA Oxoid: 18–28 mm, YES agar (Yeast Extract Sucrose agar): 28–42 mm, OAT agar (oatmeal agar): 20–34 mm, CYA at 37 °C: no growth, CREA agar (Creatine sucrose agar): 13–19 mm, weak growth, no acid production. Colonies on CYA floccose, moderate to good sporulation, reverse dark orange brown to dark brown, conidia *en masse* dull green to grey green, exudate droplets produced, large, light yellow, colonies on MEA, moderate to good sporulation, colony reverse orange to orange brown, the colour diffusing into the agar, colonies on YES moderate sporulation, reverse red brown to violet brown, colonies on oat meal agar, good sporulation, reverse yellow to orange. Pictures of colonies grown on CYA, YES and MEA are shown in Fig. 6.

##### Micromorphology

Penicilli irregularly biverticillate and strongly divaricate, borne from aerial hyphae, often appearing as a divergent tetrad of metulae, but also bearing intercalary metulae and even a lower ramus with metulae or very irregular, some metulae appearing as monoverticillate independent penicilli, stipes varying in length and character, mostly 50–400 × 2.5–3.2 μm, smooth-walled, metulae cylindrical to slightly apically swollen, 8–16 μm × 2–3 μm, phialides ampulliform in verticils of 5–8, 6.5–8 μm × 2.2–2.8 μm with a distinct neck, conidia globose, smooth to finely roughened wall ornamentation, arranged in poorly defined columns. Photomicrographs of conidiophores and conidia are shown in Fig. 6.

## Methods

### Strain isolation and preservation

The isolates IBT 12285, IBT12287 and IBT12289 were obtained from soil in Grand Canyon, south Rim, USA, Arizona in July 1990. The cultures were contained in the culture collection of DTU, Department of Systems Biology, Denmark (IBT). The strains have also been deposited at CBS (see under description of the new species). Strain IBT 12289 was used for genome sequencing and for this study.

### Genomic DNA extraction

*P. arizonense* was grown for 7 days at 25 °C on CYA agar to induce sporulation. Conidiophores were harvested in water with 0.1% tween (v/v) and 0.9% NaCl (w/v), and used for inoculation (10^9^ spores/l) of 500 ml baffled Erlenmeyer shake flasks with a working volume of 150 ml CYA medium. Cultivation was carried out for 5 days at 25 °C with orbital shaking (150 rpm). Mycelium was isolated from the remaining culture broth by filtration through mira-cloth and subsequently lyophilized. Extraction of genomic DNA for sequencing is described in Supplementary Methods S1.

### Genome sequencing and assembly

Extracted DNA was sequenced using illumina 2500 technology, yielding 125 bp paired end reads with an average insert size of 350 bp. The raw fastq files were quality checked and assembled with various assembly tools (ABySS^84^ (v 1.5.2), SOAPdenovo (v. 2.04), SPAdes (v. 3.5.0) and MIRA (v. 4.0.2)). De bruijn graph based assemblers were tested with k-mer values between 55–117, and the quality of the assemblies was assessed using QUAST^85^ and FRCalign^86^. Finally the assembly of ABySS with a k-mer size of 93 was chosen as the best assembly and was used for the further analysis.

### Genome annotation

A custom gene prediction pipeline using the Maker^87^ package was applied to combine evidence data (protein homology, transcripts, repeats) and *ab initio* predictions into gene annotations (for details see Supplementary Methods S2). Predicted genes were functionally annotated based on the protein sequence queried against Swiss-prot to retrieve gene name and protein functions following the best-hit principle when the BLAST e-value was inferior to 1e-6. In addition, tRNAscan^88^ (v. 1.3.1) was used to predict tRNAs. To identify the mitochondrial genome, a nucleotide blast of the genome assembly against the mitochondrial genome of *P. solitum*^48^ was conducted to unambiguously identify one highly similar contig most likely representing the mitochondrial genome of *P. arizonense*. Mitochondrial genes were annotated using the MFannot tool (http://megasun.bch.umontreal.ca/cgi-bin/mfannot/mfannotInterface.pl).

For a functional comparison of the predicted proteome of *P. arizonense* we downloaded the proteomes of 6 related species and classified all proteins into KOGs (retrieved on 2015-08-21 from: ftp.ncbi.nih.gov/pub/mmdb/cdd/little_endian) using rpsblast, with an e-value cut-off set to 0.01 (see list of species in Supplementary Table S1). Proteins of each species were subsequently mapped to the CAZy database using family specific profile hidden markov models, downloaded from dbCAN^89^ (v. 4.0). For each CAZyme, secretion was predicted by evaluating the presence of a secretion signal sequence using signalP^90^ (v. 4.1) with default parameters.

Secondary metabolite biosynthetic gene clusters were predicted using antiSMASH^56^ (v. 3.0.4) in *P. arizonense* and related *Penicillium* genomes for comparison (see list of species in Supplementary Table S2). Predicted gene clusters in *P. arizonense* were connected to known pathways based on the antiSMASH output and custom BLAST analysis. Two genes where considered orthologous if a protein BLAST resulted in a global identity of more than 30% and a coverage more than 50%.

### Phylogenetic analysis

The phylogenetic relationship within section *Canescentia* and related species was inferred based on partial sequences of the genes RPB2 (602 bp), CaM (427 bp). BenA (410 bp), as well as the Internal Transcribed Spacer sequence (ITS) (522 bp). The sequences were extracted with accession numbers from NCBI (see Supplementary Data S3), and the corresponding loci in the *P. arizonense* genome were identified with a nucleotide BLAST using the sequences of related species as query. A nucleotide multiple sequence alignment was generated using MUSCLE^91^ (v. 3.8.31) with default parameters and subsequently, poorly aligned regions were removed using Gblocks^92^ (v. 0.91b) with parameters -b2 = 50 -b5 = a, and concatenated into a single sequence of 1961 nucleotide positions. A maximum likelihood phylogenetic tree was inferred using PhyML^93^ (v. 20120412), with GTR + I + G as substitution model. Support in nodes was calculated with 1000 bootstrap replicates and only bootstrap support of more than 80% is shown.

### Secondary me**t**abolite analysis

The cultures were grown on CYA (Czapek Yeast Autolysate agar: 5 g/l yeast extract, 35 g/l Czapek dox broth, 1 ml/l trace metal solution, 15 g/l agar), YES (Yeast Extract Sucrose agar, 20 g/l yeast extract, 150 g/l sucrose, 0.5 g/l MgSO_4_·7H_2_O, 1 ml/l trace metal solution, 15 g/l agar), OAT (30 g/l oatmeal, 1 ml/l trace metal solution, 15 g/l agar), for 7 days at 25 °C. Three agar plugs were sampled from one colony on each medium and 1.0 ml of extraction solvent, isopropanol:ethylacetate (1/3) containing 1% formic acid, was added. After ultra-sonification for 1 h the extract was transferred to a clean vial, evaporated to dryness and redissolved in 100 μl methanol. After centrifugation for 5 min the supernatant was directly used for chemical analysis.

Secondary metabolite profiling was done by UHPLC-DAD-TOFMS on a maXis HD orthogonal acceleration quadrupole time-of-flight mass spectrometer (Bruker Daltonics, Bremen, Germany) equipped with an electrospray ionization (ESI) source and connected to an Ultimate 3000 UHPLC system (Dionex, Thermo Scientific, Dionex, Sunnyvale, CA). The column used was a Kinetex 2.6 μmC18, Ā 00D7 2.1 mm (Phenomenex, Torrance, CA) maintained at 40 °C with a flow rate of 0.4 ml/min. A linear gradient system composed of 20 mmol/L formic acid in water, and 20 mmol/L formic acid in acetonitrile was used, starting from 10% (v/v) acetonitrile and increased to 100% in 10 min, maintaining this rate for 3 min before returning to the starting conditions within 0.1 min and maintaining these for 2.4 min before the following run. TOFMS was performed in ESI^+^ with a data acquisition range of 10 scans per second at m/z 100–1,000, switching between 0 and 20 eV fragmentation energy. The TOFMS was calibrated at the start of each analytical run using Bruker Daltonics high precision calibration algorithm with the use of the internal standard sodium formate. UV/Vis spectra were collected at wavelengths from 200 to 700 nm. Identification of secondary metabolites was performed using aggressive dereplication of the full HRMS data, and pseudo MS/MS data from the 20 eV fragmentation trace, using a search list of compounds based on former taxonomic identification and a manual search of major peaks in the internal library. This library consists of 1500 compounds of which 95% are fungal secondary metabolites^94^. Compounds were confirmed by comparison of HRMS, UV/Vis and MS/HRMS to a reference standard.

True tandem MS/HRMS spectra were made on an Agilent 1290 UHPLC system (Agilent Technologies, Torrance, CA) using a similar separation system and coupled to an Agilent 6545 QTOF where MS/HRMS spectra were obtained at fixed collision-induced dissociation (CID) energies of 10, 20, and 40 eV^94^ and matched to the internal library of approx. 1500 reference standards and previously tentatively identified compounds.

### Morphological analysis

Colony descriptions were based on 7 days growth of cultures CYA agar, Blakeslee malt extract agar (MEA), Oxoid malt extract agar (MEA-Ox), YES agar, OAT agar, and creatine sucrose agar incubated in darkness at 25 °C^1^. Micromorphological features were examined on MEA.

## Additional Information

**Accession codes:** The *Pencillium arizonense* whole genome shotgun project has been deposited at DDBJ/ENA/ GenBank under the accession LXJU00000000. The version described in this paper is version LXJU01000000.

**How to cite this article**: Grijseels, S. *et al.* *Penicillium arizonense*, a new, genome sequenced fungal species, reveals a high chemical diversity in secreted metabolites. *Sci. Rep.* **6**, 35112; doi: 10.1038/srep35112 (2016).

## Supplementary Material

Supplementary Information

Supplementary Dataset

## Figures and Tables

**Table 1:** General features of the *P. arizonense* genome assembly.

Nuclear genome	
Size of assembled genome (Mb)	33.7
GC content (%)	49.1
Number of genes	12,502
Number of genes with introns	10,083
Mean exon per gene	3
Mean intron per gene	2
Longest gene (bp)	18,858
Mean gene length (bp)	1,614
Mean exon length (bp)	448
Mean intron length (bp)	95
Assembled genome covered by genes (%)	59.8
Number of tRNA genes	182
**Mitochondrial genome**	
Size of mitochondrial genome (bp)	28,347
GC content (%)	25
Number of genes	43
Number of tRNA genes	24

**Table 2:** Summary of secondary metabolites identified in extracts of *P. arizonense* and predicted biosynthetic gene clusters.

Detected compound	Most similar biosynthetic gene cluster	Orthologous genes^b^	Avg. similarity [% ID/% coverage]
Austalide B, J^a^, K, L, novel isomers C_25_H_32_O_8_ and C_26_H_34_O_9_	Cluster 4: Similarity to a mycophenolic acid cluster^58^	3/8	59/96
6-Farnesyl-5-7-dihydroxy-4-methylphthalide^a^			
Pyripyropene A^a^, E, F, O	Cluster 5: Similarity to a pyripyropene cluster^45^	7/9	79/99
Tryptoquivaline C^a^ or 27-epi-Tryptoquivaline^a^, G or L, I, M or 27-epi-Nortryptoquivaline	Cluster 3: Similarity to a tryptoquialanine cluster^67^	13/13	74/96
Fumagillin^a^	Cluster 34: Similarity to an intertwined fumagillin/pseurotin cluster^71^,^95^	16/16	82/95
Pseurotin A^a^			
Xanthoepocin	Cluster 2: Similarity to an aurofusarin cluster^79^,^80^	7/11	49/96
Curvulinic acid^a^	n.d.	n.a.	n.a.
n.d.	Cluster 28: Similarity to an acetylaranotin cluster^83^	9/9	68/91

All compounds were found in extract of *P. arizonense* IBT 12295, 12287 and 12289. ^a^confirmed with a standard.

^b^Number of genes in the predicted *P. arizonense* gene cluster which are orthologous (more than 30% identity and 50% coverage) to a gene in the reference gene cluster. For detailed information about the detected gene clusters see Supplementary Data S1 and S2.
