# Supplementary material for: Penicillium arizonense, a new, genome sequenced fungal species, reveals a high chemical diversity in secreted metabolites
Source: Sci Rep. 2016 Oct 14;6:35112. doi: 10.1038/srep35112 (PMC5064400; doi:10.1038/srep35112)
Supplement: Supplementary Information [file srep35112-s1.pdf]

Supplementary information

Penicillium arizonense, a new, genome sequenced fungal species, reveals a high chemical diversity in secreted metabolites

Sietske Grijseels[1¤], Jens Christian Nielsen[2¤], Milica Randelovic[1], Jens Nielsen[2,3], Kristian Fog Nielsen[1], Mhairi Workman[1], Jens Christian Frisvad[1]*

[1] Department of Systems Biology, Technical University of Denmark, DK2800 Kgs. Lyngby, Denmark
[2] Department of Biology and Biological Engineering, Chalmers University of Technology, SE41296 Gothenburg, Sweden
[3] Novo Nordisk Foundation Center for Biosustainability, Technical University of Denmark, DK2800 Kgs. Lyngby, Denmark
¤ These authors contributed equally to the work
* Corresponding author: Department of Systems Biology, Building 221, Soltofts Plads, Technical University of Denmark, 2800 Kgs. Lyngby, Denmark. Tel: +45 4525 2626, Fax.: +45 45884922, E-mail: jcf@bio.dtu.dk

Supplementary Table S1: Genome statistics of P. arizonense and 6 related fungi. [a]Accessed at NCBI (http://www.ncbi.nlm.nih.gov/). [b]Accessed at AspGD (http://www.aspgd.org/.)

Species	Strain	Genome size (Mb)	# genes	GC content (%)	Reference
Penicillium arizonense[a]	CBS 141311	33.7	12502	49.1	This study
Aspergillus nidulans[b]	FGSC A4	30.5	10776	49.2	1
A. niger[b]	CBS 513.88	34.0	14069	50.3	2
A. oryzae[b]	RIB40	37.9	12090	47.2	3
P. oxalicum[a]	114-2	30.2	9979	50.6	4
P. rubens[a]	Wis. 54-1255	32.2	13671	48.9	5
Trichoderma reesei[a]	QM6a	33.4	9115	52.7	6

Supplementary Table S2: AntiSMASH predictions of secondary metabolite biosynthetic gene clusters in P. arizonense and other genome sequenced Penicillium species. All genomes were downloaded from NCBI (http://www.ncbi.nlm.nih.gov/).

Species	PKS	NRPS	other	terpene	hybrid	siderophore	indole	Total
P. expansum[7]	22	16	10	7	13	0	2	70
P. camemberti[8]	21	12	9	15	6	0	3	66
P. griseofulvum[9]	22	11	14	5	8	0	3	63
P. arizonense	**28**	**16**	**8**	**5**	**3**	**1**	**1**	**62**
P. rubens	18	7	15	7	5	1	0	53
P. brasilianum[10]	16	10	11	6	4	0	1	48
P. oxalicum	7	10	10	7	7	1	2	44
P. italicum[7]	17	7	6	6	4	0	0	40
P. roqueforti[8]	10	5	7	9	6	1	1	39
P. digitatum[9]	11	11	6	6	3	0	0	37

Supplementary Table S3: Molecular formula, retention time (RT), and peak area of $[M+H]^+$ of compounds detected for P. arizonense grown on CYA, YES and OAT in duplicates. All data is based on the data from the Bruker MaXis UHPLC-DAD-TOFMS system.

Compound	Molecular formula	RT	Peak area		Peak area		Peak area	
			CYA sample 1	CYA sample 2	YES sample 1	YES sample 2	OAT sample 1	OAT sample 2
Austalide B	C26H34O8	7.43	2449513	2367205	3200473	2839876	1314724	211335
Austalide J*	C25H32O7	6.38	2017590	1558810	2139264	1547265	1573091	248092
Austalide K	C25H32O5	8.36	134911	178629	155439	126315	82372	18867
Austalide L	C25H32O6	7.35	793830	1181318	1053715	997386	458372	107905
Austalide novel isomer C25H32O8	C25H32O8	5.55	3220677	3743813	4917709	3986094	3142541	603295
Austalide novel isomer C26H34O9	C26H34O9	5.7	2116729	2152282	5930904	5356297	3552312	559506
Austalide novel isomer C26H34O9	C26H34O9	6.03	78699	73932	232652	204056	129657	22165
6-Farnesyl-5-7-dihydroxy-4-methylphthalide	C24H32O4	9.78	183567	208367	137262	124304	44476	
Pyripyropene A*	C31H37NO10	6.33	8405222	8096618	8943991	8970599	5780343	920746
Pyripyropene E	C27H33NO5	7.91	5922722	6254227	2871628	3049951	851256	165856
Pyripyropene F	C28H35NO5	8.55	165131	262361	88308	104289	16667	
Pyripyropene O	C29H35NO7	7.04	7920167	7976802	4856508	5335870	1807925	285732
Tryptoquivaline/C*/27-epi-Tryptoquivaline	C29H30N4O7	7.82	7537755	7881666	6719074	6533518	3047515	435139
Tryptoquivaline G/L	C23H20N4O5	5.94	452375	545913	530890	580052	55764	
Tryptoquivaline I	C27H26N4O6	8.23	3386415	3932047	3959818	4046346	320145	60319
Tryptoquivaline M/27-epi-Nortryptoquivaline	C28H28N4O7	7.35	206394	264111	114769	139008	22505	
Fumagillin*	C26H34O7	7.44	67935	185155	173561	185302		
Pseurotin A*	C22H25NO8	4.11	152851	317973	41543	38567		
Xanthoepocin	C30H22O14	7.14	128099	159509	316878	376334	142868	29432
Curvulinic acid*	C10H10O5	1.85	310208	561851	168229	180706	15072	

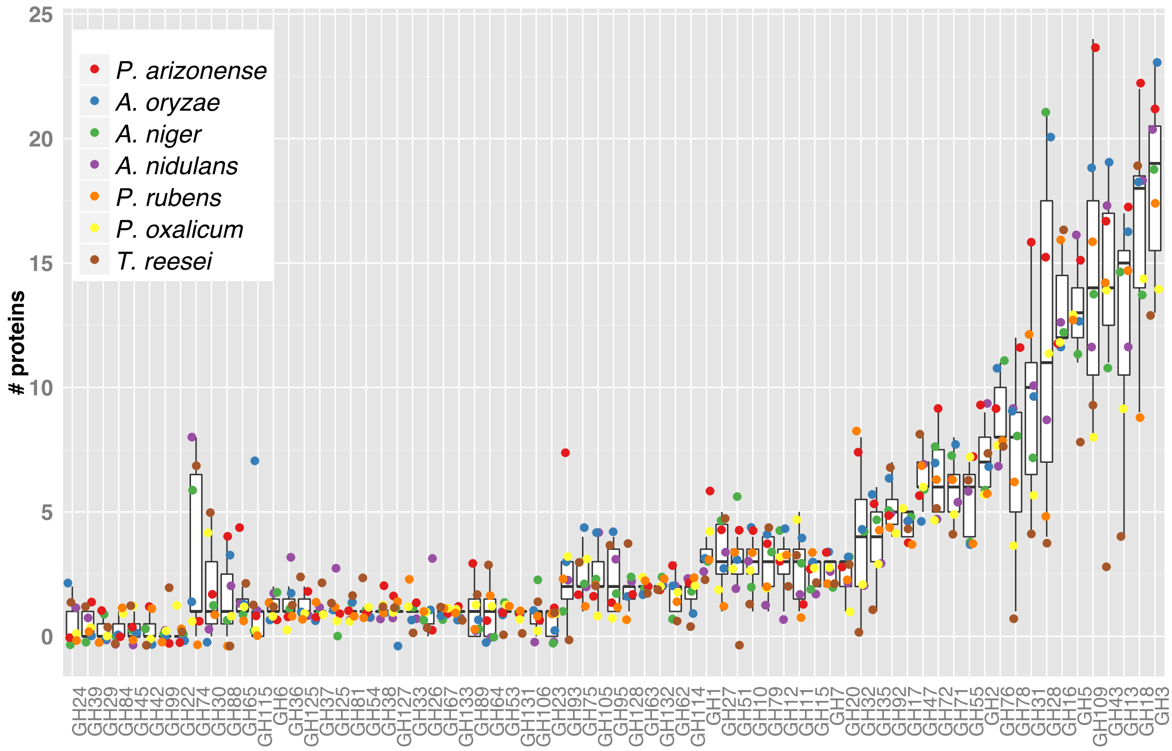

Supplementary Figure S1: Distribution of glycoside hydrolase (GH) families in P. arizonense and 6 related fungi. Within GH families 1,65 and 93, P. arizonense had a considerably higher number of proteins than the compared species.

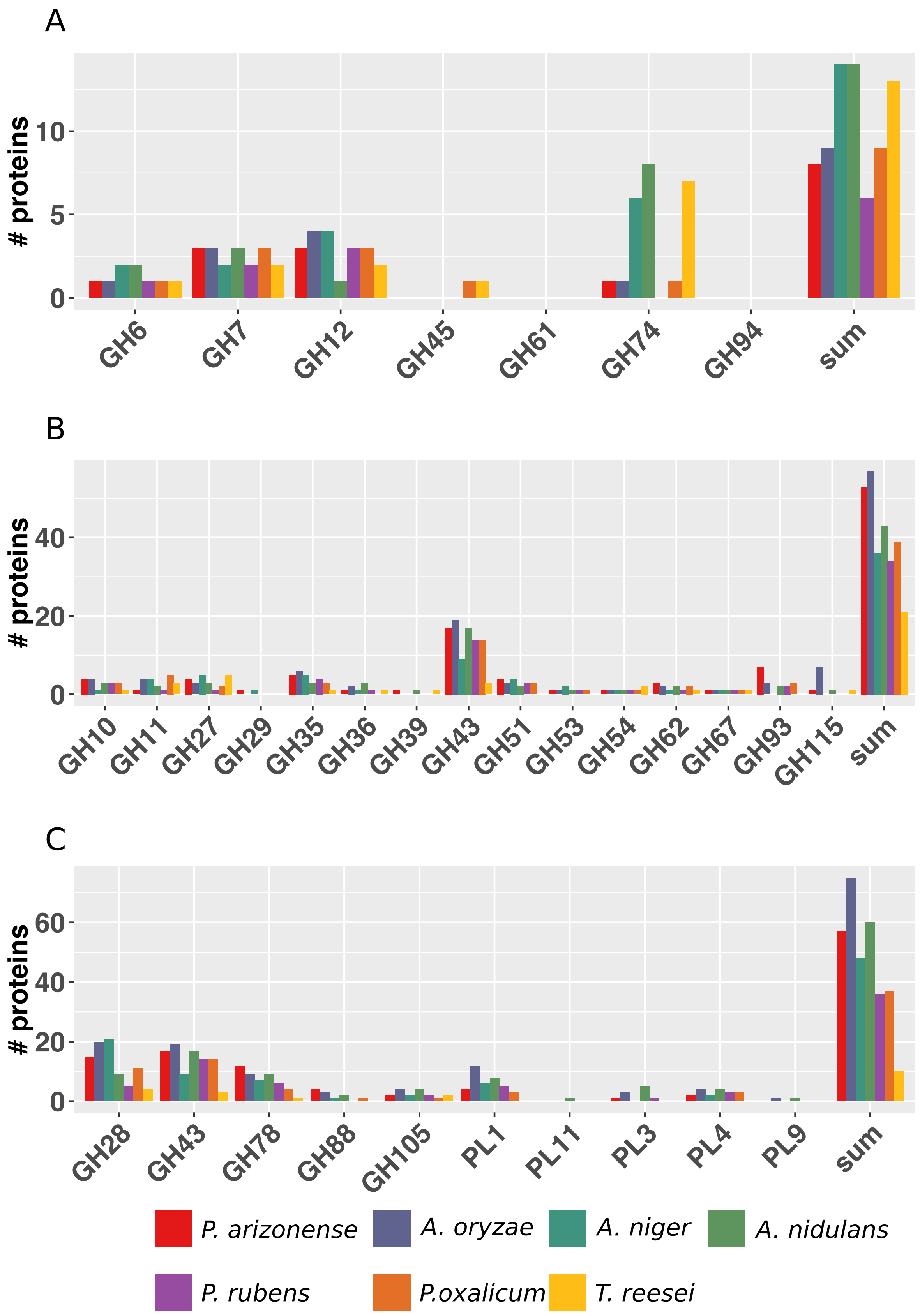

*Supplementary Figure S2: Distribution of carbohydrate active enzymes (CAZys) with plant cell wall degrading activities encoded in the genomes of P. arizonense and 6 related fungi. (**A**) CAZys with cellulase activity, (**B**) CAZys with hemi-cellulase activity and (**C**) CAZys with pectinase activity. Compared to related species, P. arizonense proved to posses a high number of CAZys with hemi-cellulase activity, a low number of CAZys with cellulase activity, while the number of CAZys with pectinases activity was among the highest.*

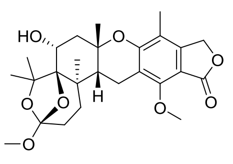

Austalide B

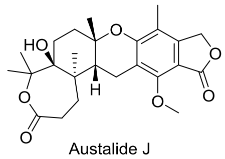

Austalide J

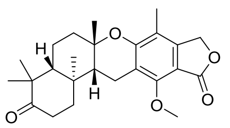

Austalide K

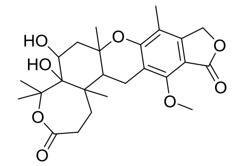

Austalide isomer C25H32O8

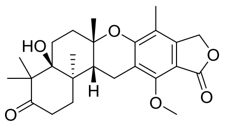

Austalide L

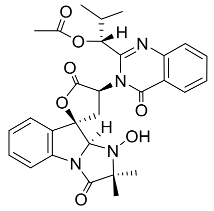

Tryptoquivaline C

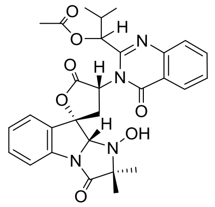

27-epi-Tryptoquivaline

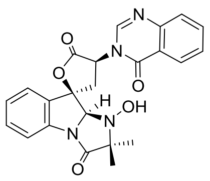

Tryptoquivaline G

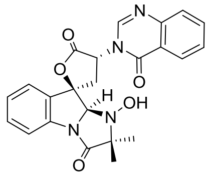

Tryptoquivaline L

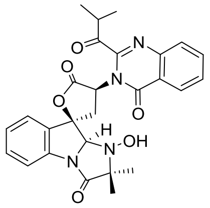

Tryptoquivaline I

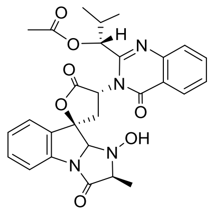

Tryptoquivaline M

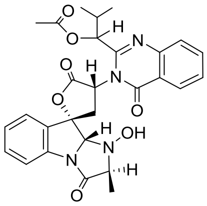

27-epi-Nortryptoquivaline

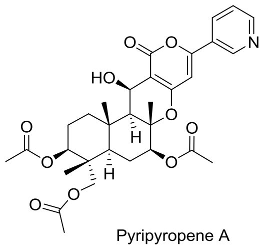

Pyripyropene A

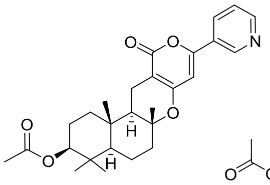

Pyripyropene E

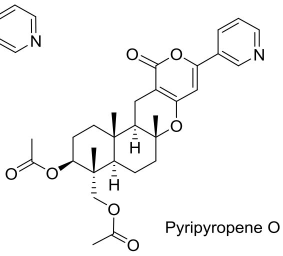

Pyripyropene O

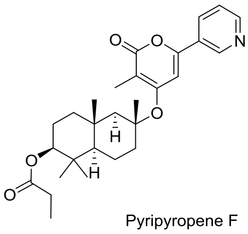

Pyripyropene F

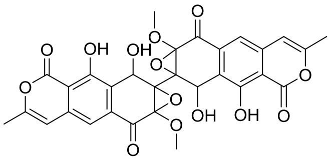

Xanthoepocin

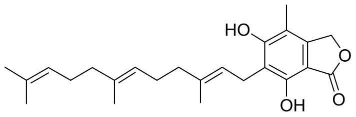

6-Farnesyl-5-7-dihydroxy-4-methylphthalide

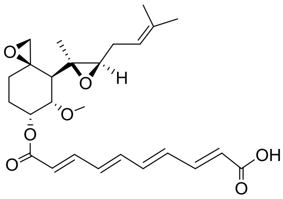

Trans-fumagillin

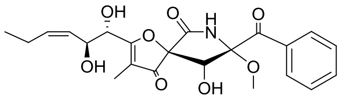

Pseurotin A

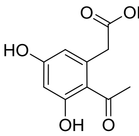

Curvulinic acid

Supplementary Figure S3: structures of compound detected in this study. Structures of tryptoquivaline L, M, G and C from Yamazaki et al. (1979)[11], tryptoquivaline G from Yamazaki et al. (1978)[12] and 27-epi-nortryptoquivaline from Fujimoto et al. (1996)[13].

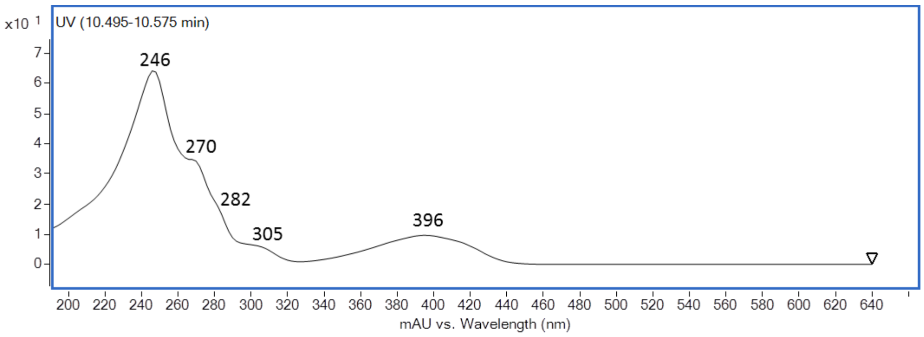

Supplementary Figure S4: Table UV spectrum of xanthoepocin. The maxima fit very well with the maxima according to Igarashi et al.[14] in acidic conditions: 246 (4.76), 268 (4.52), 282 (sh), 306 (sh), 393 (4.02).

Supplementary Methods S1

Extraction of high quality DNA for Genome Sequencing

Reagents Required

Buffer A: 0.35 M sorbitol
0.1 M Tris-HCL, pH 9
5 mM EDTA, pH 8

Buffer B 0.2 M Tris-HCL, pH 9
50 mM EDTA, pH 8
2 M NaCl
2 % CTAB

5 % Sarkosyl
5 M Potassium Acetate (KAc), pH 7.5
3 M Sodium Acetate (NaAc)

Buffer A – 500ml	
3.5M Sorbitol	31,9g
1M Tris-HCl	50mL
0.5M EDTA	5mL
Buffer B – 500ml	
1M Tris-HCl	100mL
0.5M EDTA	50mL
NaCl	58,44g
CTAB	

1 % PVP
Phenol:Chloroform:Isoamylalcohol (25:24:1)
100 % Isopropanol
70% Ethanol (Ice cold)
99% Ethanol (Ice cold)
RNAse A (100 mg/ml)
Proteinase K (20 mg/ml)

Protocol

- Pre-heat Buffer B at 65 °C
- Prepare Lysis Buffer just before use. For 10 ml use:
  - 3.75 ml Buffer A
  - 3.75 ml Buffer B
  - 1.5 ml 5 % Sarkosyl
  - 1 ml 1 % PVP
  - 100 μl Proteinase K
- Grind freeze-dried mycelia in a mortar cooled with liquid nitrogen and transfer it to a 50 ml Falcon tube, with powder just below the 5ml mark
- Add 10 ml Lysis Buffer and mix vigorously by vortexing
- Incubate 30 min at 65 °C, mix frequently by inverting the tube
- Add 3.35 ml KAc (5 M), mix by inverting the tube and incubate 30 min on ice
- Centrifuge for 30 min at 5,000 g at 4 °C
- Transfer the supernatant, approximately 9mL, to a new 50 ml Falcon tube and add 5ml of Phenol:Chloroform:Isoamylalcohol (25:24:1)
- Centrifuge 20 min at 4,000 g at 4 °C
- Transfer the aqueous phase (~8mL) to a new 50 ml Falcon tube
- Add 100 μl RNAse A (10 mg/ml) – incubate at room temp. for 10-60 min.
- Add 1/10 vol of NaAc (3 M) and 1 vol of Isopropanol (RT) and incubate 5 min at room temperature
- Centrifuge for 30 min at 10,000 g at 4 °C

- Discard the supernatant
- Wash the pellet with 2 ml 70 % ethanol and centrifuge for 10 min at 10,000 g at 4 ^{o}C
- Discard the supernatant and dry the pellet at RT until all ethanol has evaporated – do not let it dry out
- Redissolve the pellet in 600 μL TE at 65 ^{o}C and transfer to 1.5 ml Eppendorf tube, make a FF=10 dilution and run gel to estimate the quality and concentration

Supplementary Methods S2

Gene prediction

Repeat regions were annotated and to increase the accuracy, we used the existing reference repeat library included in the RepeatMasker[15] (v4.0.3) enhanced by novel repeats detected with the RepeatModeler[16] package (v1.0.8). Candidate repeat sequences identified were vetted against protein sequences from the manually curated part of Uniprot[3] called Swiss-prot (retrieved on 2015-01-08) to exclude any nucleotide motif stemming from low-complexity coding sequences. After augmenting this repeat library, we utilized RepeatMasker and RepeatRunner to assign repeat sequences to genomic loci.

Genes were predicted in the masked genome by firstly training an *ab initio* gene predictor using GeneMark-ET[17] (v. 4.3) which integrates RNA-seq evidence. For this we used an in-house data set of 39 samples of RNA-seq reads from related species mapped to the *P. arizonense* genome assembly using TopHat2[18] (v2.0.9) and cufflinks[19] (v2.2.1) with settings –F 0.15 and –j 0.1. Further evidence was collected at the protein level from sequences belonging to Swiss-prot (547 357 proteins). Finally, we computed an evidence-driven annotation using the evidence sequences (proteins and transcripts) and the *ab initio* algorithm GeneMark inside of the MAKER2[20] (v. 2.31.6) genome pipeline. The completeness of the resulting gene build was assessed by detecting the presence of conserved fungal genes using BUSCO[21].

References

1. Galagan, J. E. *et al.* Sequencing of *Aspergillus nidulans* and comparative analysis with *A. fumigatus* and *A. oryzae*. *Nature* **438,** 1105–1115 (2005).
2. Pel, H. J. *et al.* Genome sequencing and analysis of the versatile cell factory *Aspergillus niger* CBS 513.88. *Nat. Biotechnol.* **25,** 221–31 (2007).
3. Machida, M. *et al.* Genome sequencing and analysis of *Aspergillus oryzae*. *Nature* **438,** 1157–1161 (2005).
4. Liu, G. *et al.* Genomic and secretomic analyses reveal unique features of the lignocellulolytic enzyme system of *Penicillium decumbens*. *PLoS One* **8,** e55185 (2013).
5. van den Berg, M. A. *et al.* Genome sequencing and analysis of the filamentous fungus *Penicillium chrysogenum*. *Nat. Biotechnol.* **26,** 1161–1168 (2008).
6. Martinez, D. *et al.* Genome sequencing and analysis of the biomass-degrading fungus *Trichoderma reesei* (syn. *Hypocrea jecorina*). *Nat. Biotechnol.* **26,** 553–560 (2008).
7. Ballester, A. *et al.* Genome, Transcriptome, and Functional Analyses of *Penicillium expansum* Provide New Insights Into Secondary Metabolism and Pathogenicity. *Mol. Plant-Microbe Interact.* **28,** 232–248 (2015).
8. Cheeseman, K. *et al.* Multiple recent horizontal transfers of a large genomic region in cheese making fungi. *Nat. Commun.* **5,** (2014).
9. Banani, H. *et al.* Genome sequencing and secondary metabolism of the postharvest pathogen *Penicillium griseofulvum*. *BMC Genomics* **17,** 19 (2016).
10. Horn, F. *et al.* Draft Genome Sequence of the Fungus *Penicillium brasilianum* MG11. *Genome Announc.* **3,** e00724–15 (2015).
11. Yamazaki, M., Okuyama, E. & Maebayashi, Y. Isolation of some new tryptoquivaline-related metabolites from *Aspergillus fumigatus*. *Chem. Pharm. Bull. (Tokyo).* **27,** 1611–1617 (1979).
12. Yamazaki, M., Haruhiro, F. & Okuyama, E. Structure determination of six fungal metabolites, tryptequivaline E, F, G, H, I and J from *Aspergillus fumigatus*. *Chem. Pharm. Bull. (Tokyo).* **26,** 111–117 (1978).
13. Haruhiro, F., Negishi, E., Yamaguchi, K., Nishi, N. & Yamazaki, M. Isolation of new tremorgenic metabolites from an Ascomycete, *Corynascus setosus*. *Chem. Pharm. Bull. (Tokyo).* **44,** 1843–1848 (1996).
14. Igarashi, Y. *et al.* Xanthoepocin , a New Antibiotic from *Penicillium simplicissimum* IF05762. *J. Antibiot. (Tokyo).* **53,** 928–933 (2000).
15. Smit, A., Hubley, R. & Green, P. RepeatMasker Open-4.0. (2015). at <http://www.repeatmasker.org>
16. Smit, A. & Hubley, R. RepeatModeler Open-1.0. (2015). at <http://www.repeatmasker.org>
17. Lomsadze, A., Burns, P. D. & Borodovsky, M. Integration of mapped RNA-Seq reads into automatic training of eukaryotic gene finding algorithm. *Nucleic Acids Res.* **42,** e119 (2014).
18. Kim, D. *et al.* TopHat2: accurate alignment of transcriptomes in the presence of insertions, deletions and gene fusions. *Genome Biol.* **14,** R36 (2013).
19. Trapnell, C. *et al.* Transcript assembly and quantification by RNA-Seq reveals unannotated transcripts and isoform switching during cell differentiation. *Nat. Biotechnol.* **28,** 511–515 (2010).
20. Holt, C. & Yandell, M. MAKER2: an annotation pipeline and genome-database management tool for second-generation genome projects. *BMC Bioinformatics* **12,**

491 (2011).
21. Simão, F. A., Waterhouse, R. M., Ioannidis, P., Kriventseva, E. V. & Zdobnov, E. M. BUSCO: Assessing genome assembly and annotation completeness with single-copy orthologs. *Bioinformatics* **31,** 3210–3212 (2015).
